# Supplementary material for: Molecular mechanism of Activin receptor inhibition by DLK1
Source: Nat Commun. 2025 Jul 1;16:5976. doi: 10.1038/s41467-025-60634-3 (PMC12216052; doi:10.1038/s41467-025-60634-3)
Supplement: Supplementary file 1 — Supplementary Information [file 41467_2025_60634_MOESM1_ESM.pdf]

Supplementary Information file

Molecular mechanism of Activin receptor inhibition by DLK1

Daniel Antfolk[1], Qianqian Ming[1], Anna Manturova[1], Erich J. Goebel[2], Thomas B. Thompson[2], Vincent C. Luca[1*]

[1]Department of Immunology, Moffitt Cancer Center & Research Institute, Tampa, FL, USA.
[2]Department of Molecular and Cellular Biosciences, University of Cincinnati, Cincinnati, OH, USA

Keywords: Activin receptor, DLK1, Delta like non-canonical Notch ligand 1, Notch, transforming growth factor beta, myoblast, differentiation, myostatin

*To whom correspondence should be sent: vince.luca@moffitt.org

Supplementary information inventory:

Supplementary Figure 1-10

Supplementary Table 1-2

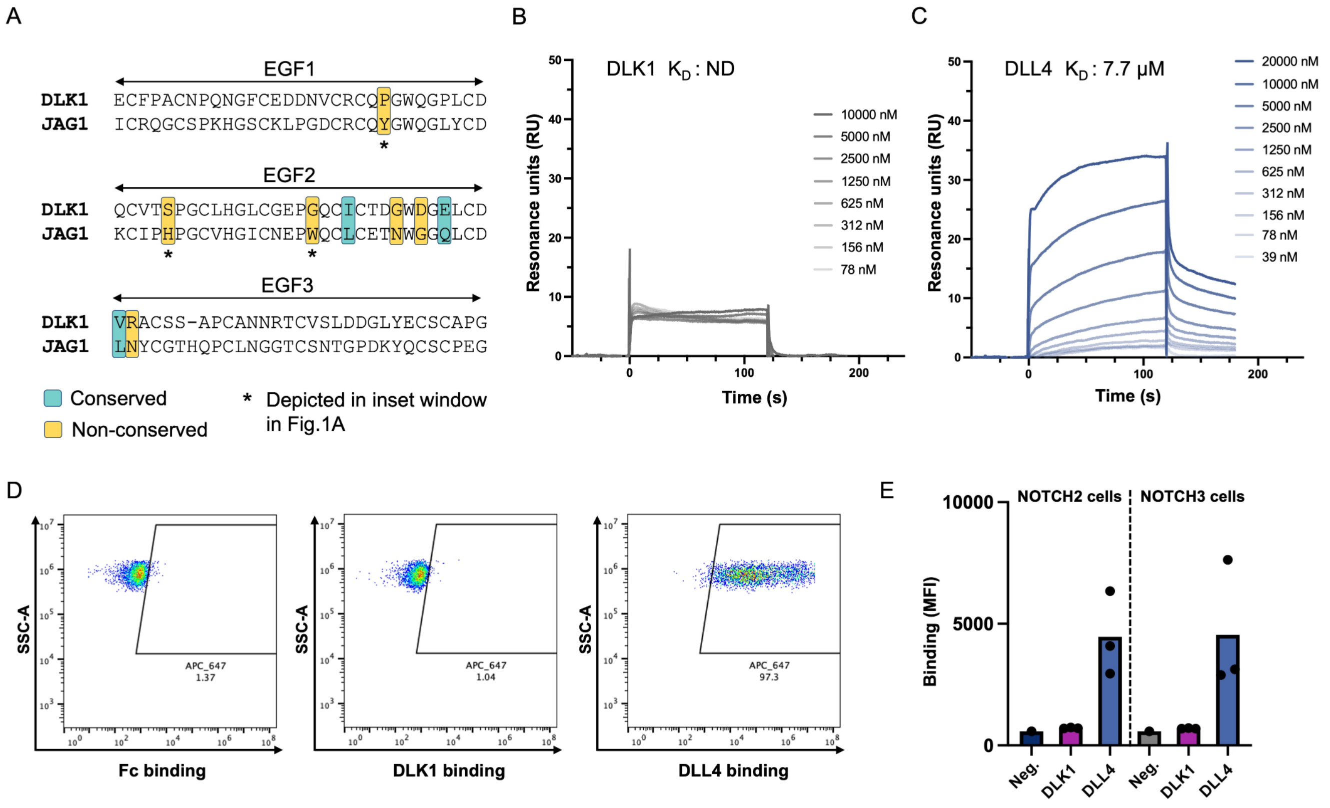

Supplementary Fig. 1. DLK1 does not bind to Notch1, Notch2, or Notch3. A Sequence alignment of the EGF1-3 regions of human JAG1 and DLK1. JAG1 residues that contact NOTCH1 and the analogous DLK1 residues are highlighted. Conserved residues are colored cyan and non-conserved residues are colored yellow. The residues marked with asterisks (Y255P*, H268S* and W280G*) are those visualized in Fig. 1A. **B-C** SPR sensograms using the extracellular domain of **B** DLK1 (N-EGF6) or **C** DLL4 (N-EGF5) as analyte with NOTCH1 (N-EGF36) immobilized on a sensor chip. RU = resonance units. **D** Flow cytometry dot plots of U2OS cells overexpressing NOTCH1 stained with Fc-tagged DLK1 or DLL4 protein with the MFI of the APC channel on the x-axis. **E** U2OS cells overexpressing NOTCH2 or NOTCH3 stained with Fc-tagged DLK1 or DLL4 protein using an anti-Fc Alexa Fluor 647 antibody as measured by flow cytometry. Bar graph depicts mean MFI based on triplicate wells for DLK1/DLL4, and one well for the unstained control cells (Neg.) from one representative experiment. The experiment was independently repeated two times. Source data are provided as a Source Data file.

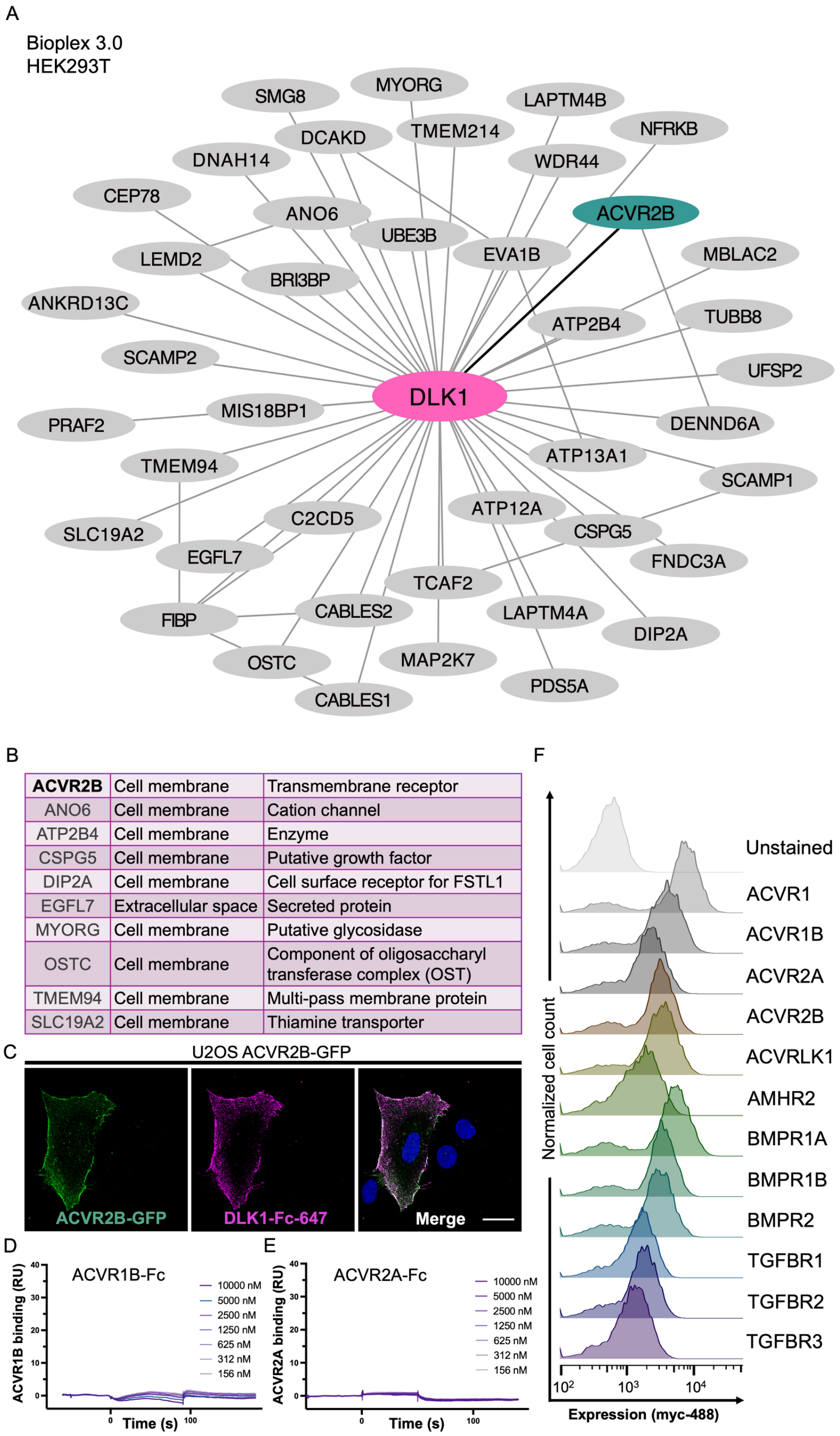

Supplementary Fig. 2. Interactome data identifies ACVR2B as a receptor for DLK1. A Bioplex 3.0 output for DLK1 depicting hits with ComPASS+ scores > 0.75, which represents the top 2% of proteins enriched in the interactome screen. **B** Gene ontology for the ten DLK1 hits including an extracellular domain or peptide. **C** U2OS cells overexpressing ACVR2B stained by DLK1-Fc protein utilizing an anti-Fc Alexa Fluor 647 antibody. Only the cell expressing ACVR2B as determined by GFP^{SPARK}-tag are stained by DLK1 in a mixed population of U2OS cells. Nuclei counterstained with DAPI (blue). Scale, 20 µm. **D-E** SPR sensograms indicating that DLK1 (N-EGF6) does not bind to immobilized ACVR1B-Fc or ACVR2A-Fc. RU = resonance units. **F** Surface expression levels of twelve yeast-displayed TGF-β superfamily receptors was determined by flow cytometry using anti c-myc Alexa Fluor 488-conjugated antibody. Source data are provided as a Source Data file.

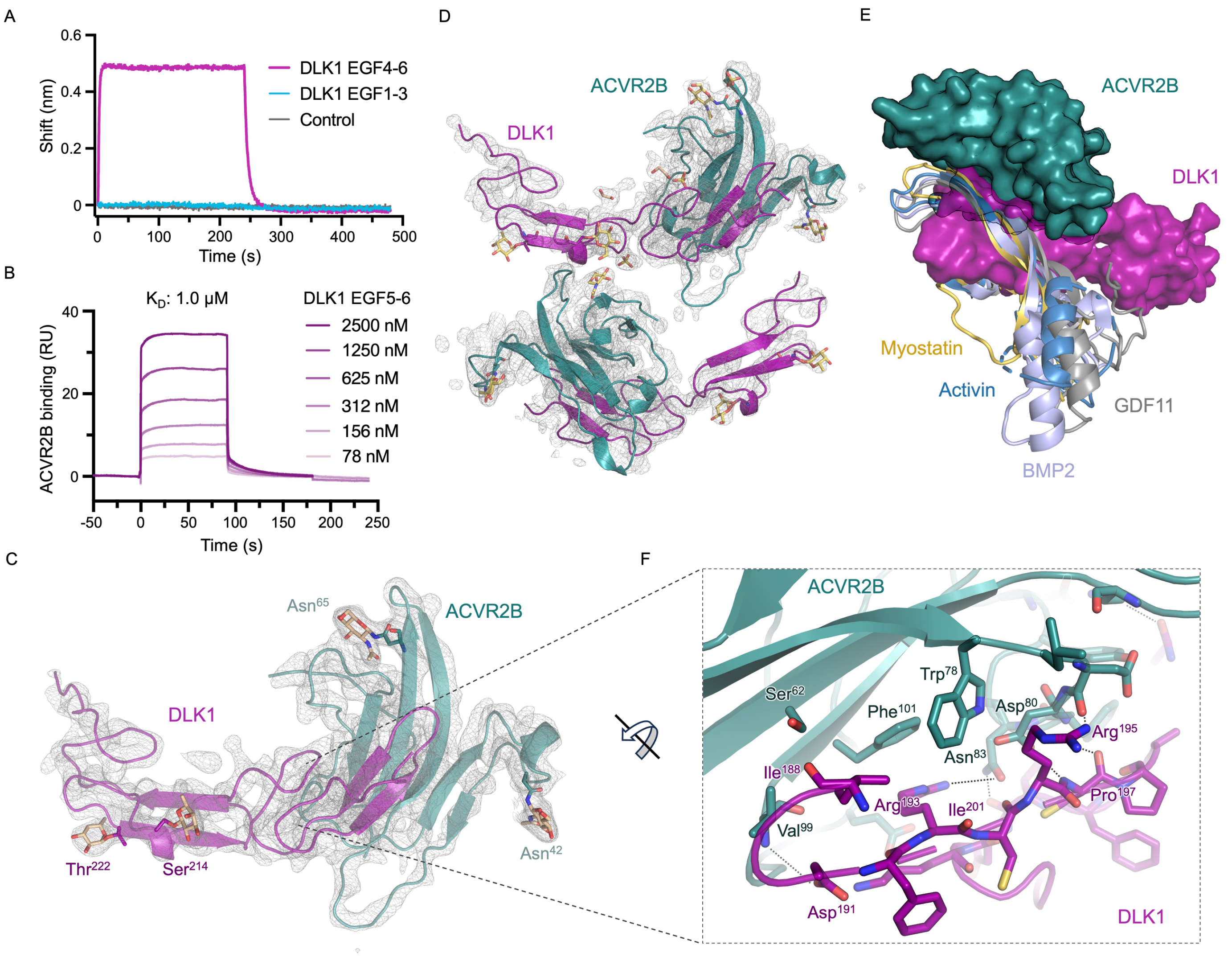

Supplementary Fig. 3. Analysis of DLK1-ACVR2B crystal structure. A Overlay of BLI sensograms depict the binding of ACVR2B (5 µM) to immobilized DLK1 EGF1-3, DLK1 EGF4-6, or control protein CD112. **B** SPR sensograms depict the binding of DLK1(EGF5-6) to immobilized ACVR2B-Fc. RU = resonance units. **C** Electron density map surrounding the DLK1(EGF5-6)-ACVR2B structure is shown with the 2Fo-Fc map contoured at 1.0 σ. Glycans on each protein are indicated, with N-linked glycans on N42 and N65 of ACVR2B, and O-linked glycans S214 and T222 of DLK1. **D** A composite omit map (2mFo-DFc) contoured at 1.2 σ. Maps were calculated using Phenix v1.13. **E** Superimposed view of the structure of DLK1 (magenta) bound to ACVR2B (teal) and structures of ACVR2B bound to canonical ligands Activin-A (blue) PDB ID: 1S4Y , GDF11 (grey) PDB ID: 6MAC and BMP2 (light purple) PDB ID: 2H64. The unliganded structure of Myostatin (yellow) PDB ID: 5JI1 was aligned with GDF11 to generate a model of the Myostatin-ACVR2B complex. **F** Zoom window depicting residues present at the DLK1-ACVR2B interface. Source data are provided as a Source Data file.

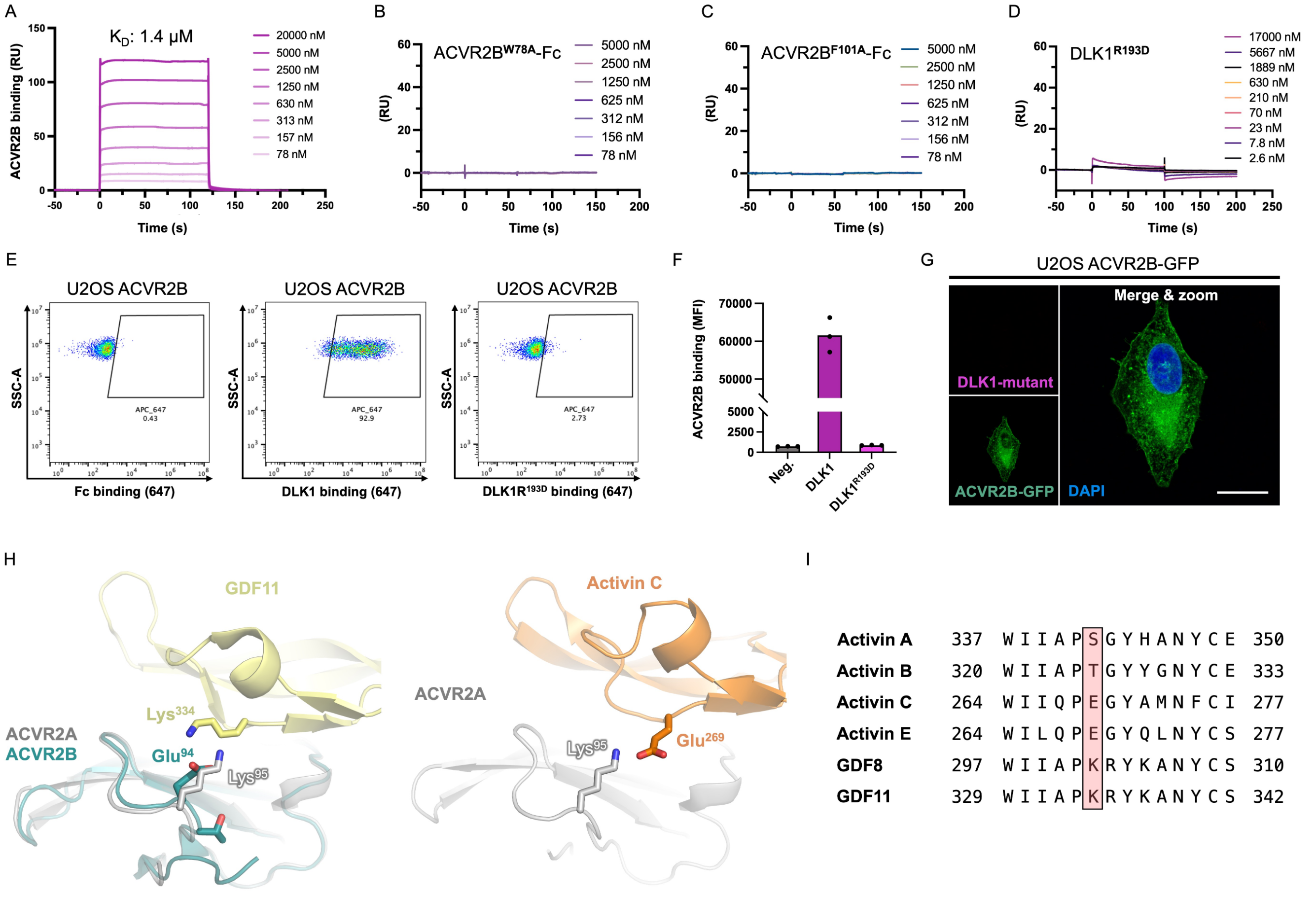

Supplementary Fig. 4. Mutational analysis of the DLK1-ACVR2B interface. Overlay plots of SPR sensograms from DLK1 ECD binding to immobilized ACVR2B-Fc (**A**), ACVR2B^{W78A}-Fc (**B**) or ACVR2B^{F101A}-Fc (**C**). **D** Overlay plot of SPR sensograms of DLK1^{R193D} leading to loss of binding with ACVR2B-Fc. RU = resonance units. **E** Dot plots of DLK1-Fc-647 and mutant DLK1^{R193D}-Fc-647 staining by flow cytometry. **F** Bar graph depicts median fluorescence intensity (MFI) of the far-red APC channel using DLK1-Fc-647 and mutant DLK1^{R193D}-Fc-647 based on triplicate wells as measured by flow cytometry from one representative experiment. The experiment was independently repeated two times. **G** DLK1 with a R193D point mutation (loss-of-ACVR2B-binding mutant) does not stain ACVR2B expressing cells. Scale, 20 µm. The experiment was independently repeated two times. **H** Structural analysis suggests that canonical TGF-β ligands may preferentially form interactions with ACVR2A or ACVR2B based on charge complementary of a key interacting residue. The model is based on the crystal structure of the GDF11: ACVR2B complex (PDB ID: 7MRZ) and an AlphaFold model of the ACVR2A: Activin C complex (ipTM = 0.77, pTM = 0.76). **I** Sequence alignment of canonical TGF-β ligands highlighting the key interacting residue to E94 of ACVR2B and K95 of ACVR2A. Source data are provided as a Source Data file.

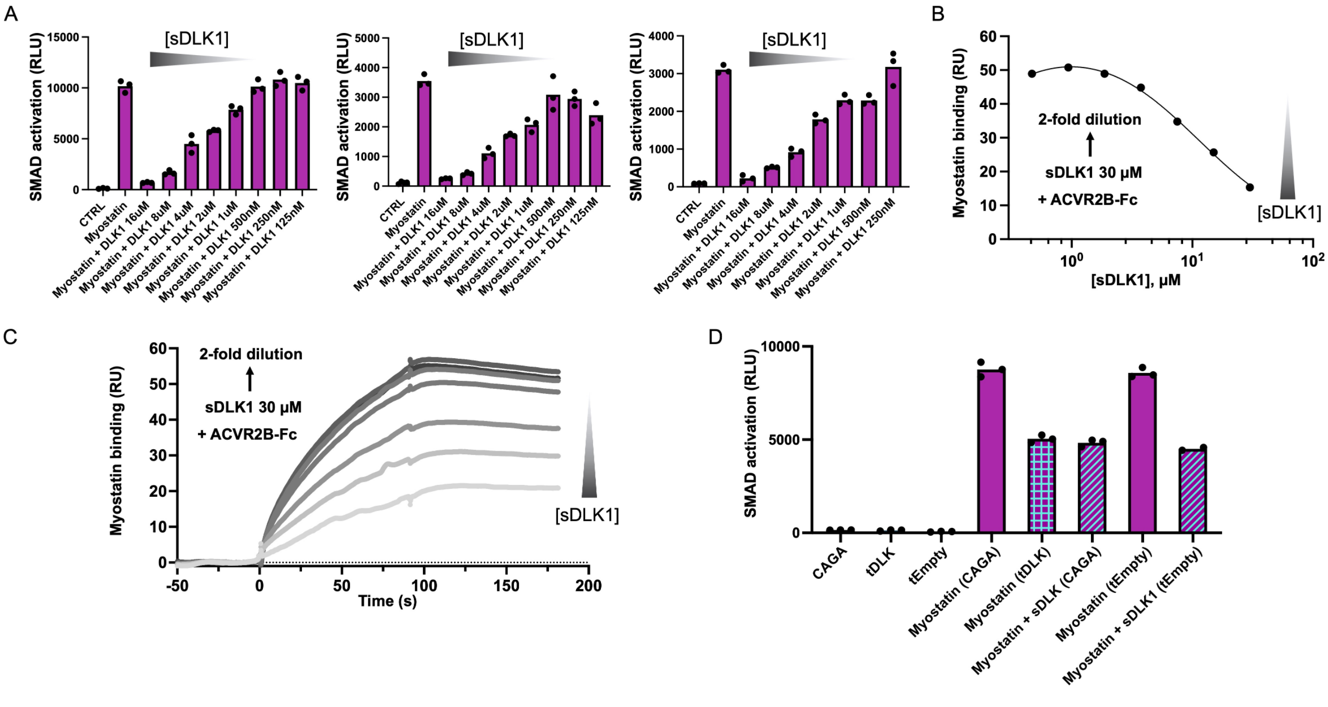

Supplementary Fig. 5. DLK1 inhibits Myostatin signaling and NICD-SMAD interactions. A Raw RLU luciferase values for three dose-titrations of DLK1 inhibiting myostatin in HEK293 SMAD-CAGA luciferase assays. Bar graph depicts relative luciferase units (RLU) based on triplicate wells from one representative experiment. The three graphs represent three independent biological replicates. The graph on the left is represented in Fig. 5B. **B** SPR was used to determine competition between DLK1 and myostatin binding to ACVR2B. ACVR2B was injected together with varying concentrations of DLK1 over a sensor chip containing immobilized myostatin. A constant concentration of 0.8 µM ACVR2B together with a 2-fold serial dilution of DLK1 starting at a concentration of 30 µM. RU = resonance units. **C** SPR sensograms used to plot the inhibition curve shown in panel B above. **D** Transfection of DLK1 into HEK293-$(CAGA)_{12}$ reporter cells inhibits Myostatin signaling. Baseline RLU values are shown for HEK293-$(CAGA)_{12}$ with or without transfected DLK1 (tDLK1), or for cells transfected with empty vector (Empty) followed by myostatin treatment at 2 nM. Inhibition by soluble DLK1 at 2.8 µM (sDLK1) was used as a comparison to transfected DLK1. Bar graph depicts RLU values as technical replicates based on one representative experiment. The experiment is an independent biological repeat of the data shown in Fig. 5C, using additional controls. Source data are provided as a Source Data file.

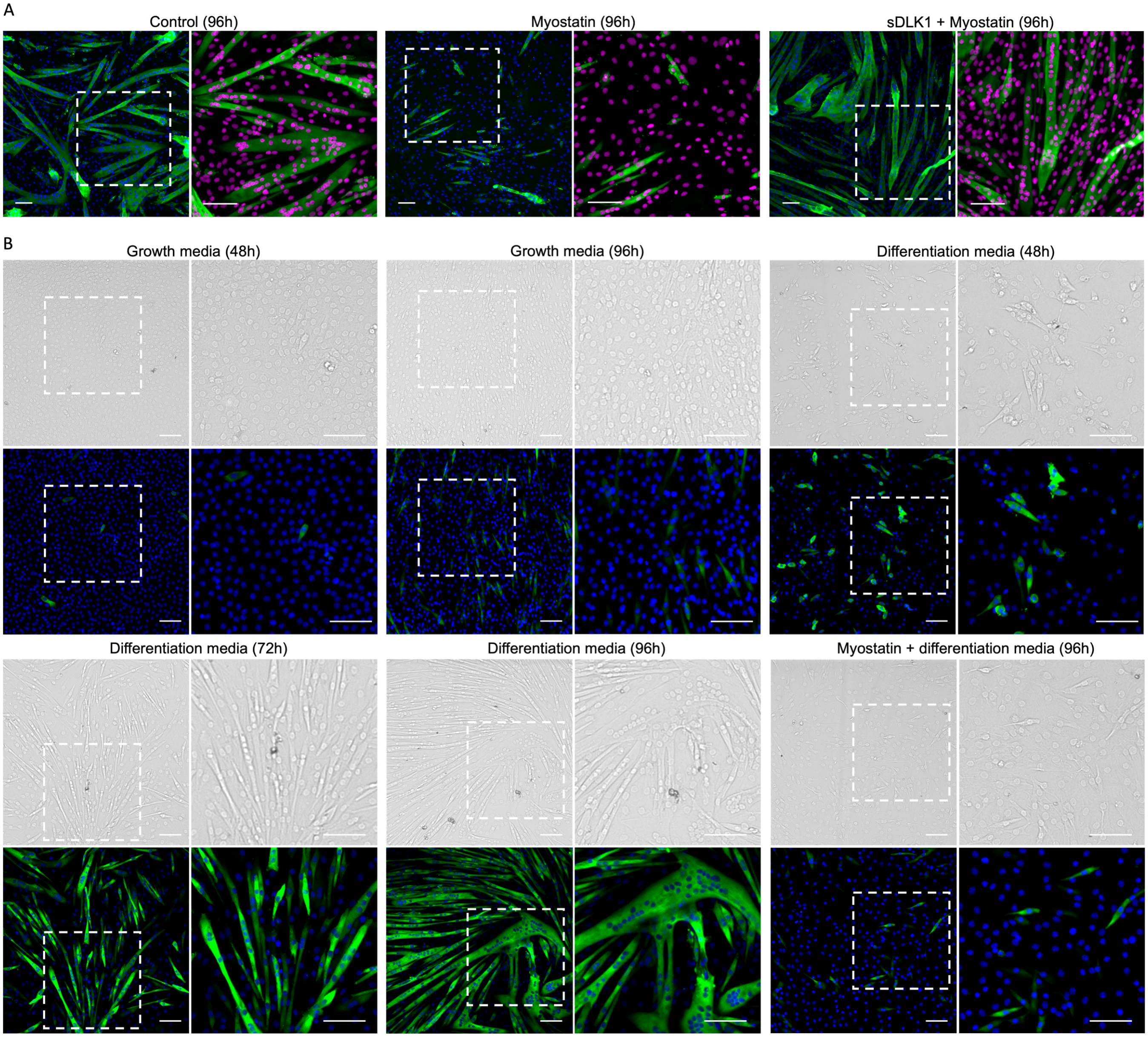

Supplementary Fig. 6. DLK1 inhibits negative effects of myostatin on C2C12 differentiation. A Representative microscopy images showing C2C12 myoblast differentiation in the presence of Myostatin or Myostatin + DLK1. Control cells were allowed to differentiate for 96h. Myostatin treatment (4 µg/ml) inhibits C2C12 myoblast differentiation into myotubes as determined by MyoHC staining. C2C12 cells were fixed with 4% PFA, immunostained with an anti-MyoHC antibody and an anti-mouse Alexa Fluor 488 secondary antibody. Nuclei were counterstained with Hoechst 33342. Nuclei represented with pseudo color (magenta) in zoom in panels. Scale bar, 100 µm. The experiment was independently repeated four times. **B** Representation of various time-points (48, 72 and 96 h) of C2C12 differentiation with comparisons of brightfield and MyoHC fluorescence at end point. Immunostaining as in A. The images are representative of one time-course optimization experiment. Scale bar, 100 µm.

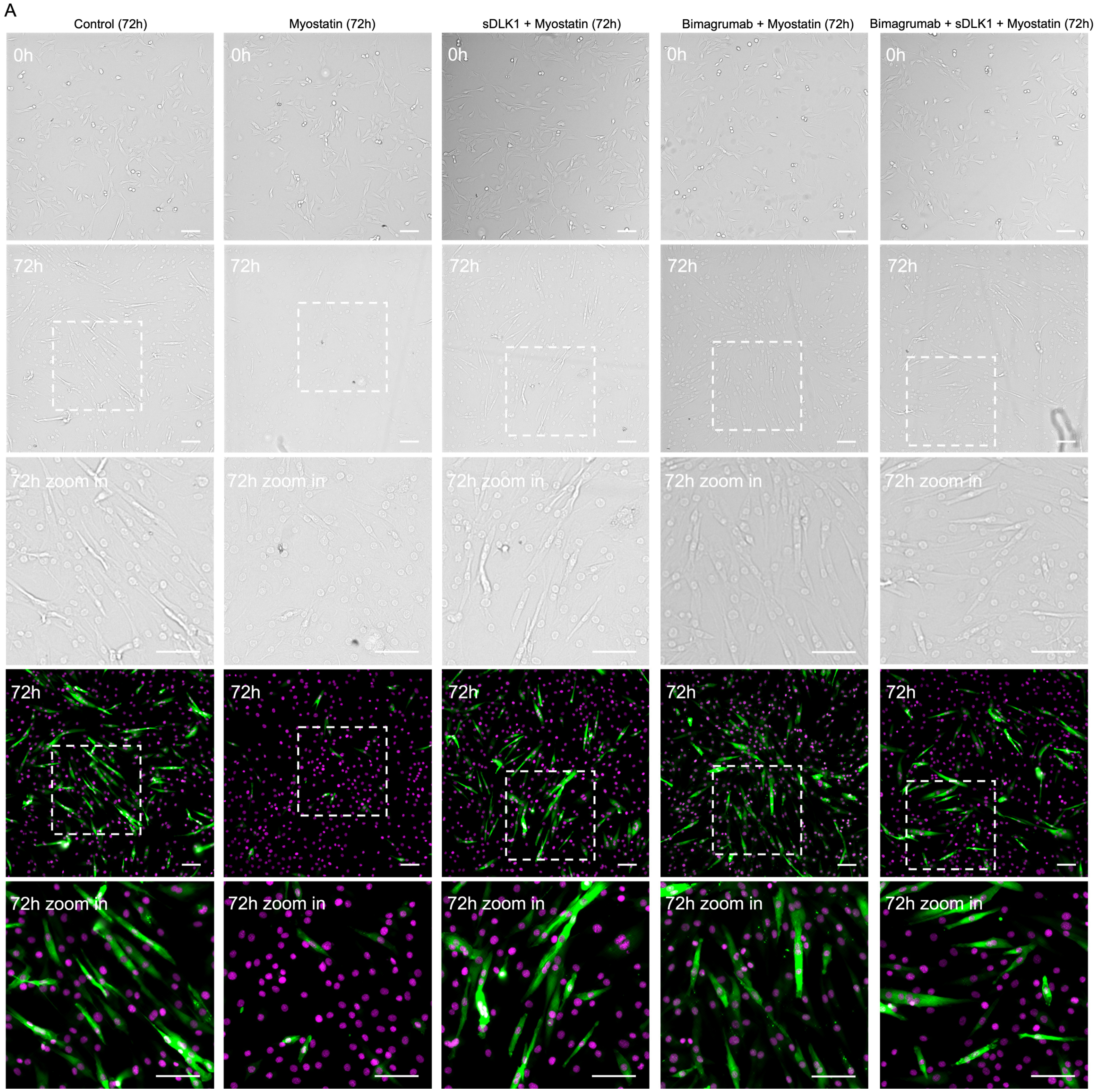

Supplementary Fig. 7. The ACVR2B antagonist antibody Bimagrumab and soluble DLK1 counteract the effects of Myostatin. A Representative microscopy images showing C2C12 myoblast differentiation in the presence of Myostatin, soluble DLK1 + Myostatin, Bimagrumab + Myostatin, or Bimagrumab + soluble DLK1 + Myostatin. Control cells were allowed to differentiate for 72h. Myostatin treatment (4 µg/ml) inhibits C2C12 myoblast differentiation into myotubes as determined by MyoHC staining. Bimagrumab (100 nM) and soluble DLK1 (2 µM) reverse the effect of myostatin on C2C12 differentiation, with no additive effect of adding soluble DLK1 to Bimagrumab treated cells. C2C12 cells were fixed with 4% PFA, immunostained with an anti-MyoHC antibody and an anti-mouse Alexa Fluor 488 secondary antibody. Nuclei were counterstained with Hoechst 33342. Nuclei represented with pseudo color (magenta). The images are representative of one out of two imaging wells per treatment from one experiment. The experiment was independently repeated two times. Scale bar, 100 µm.

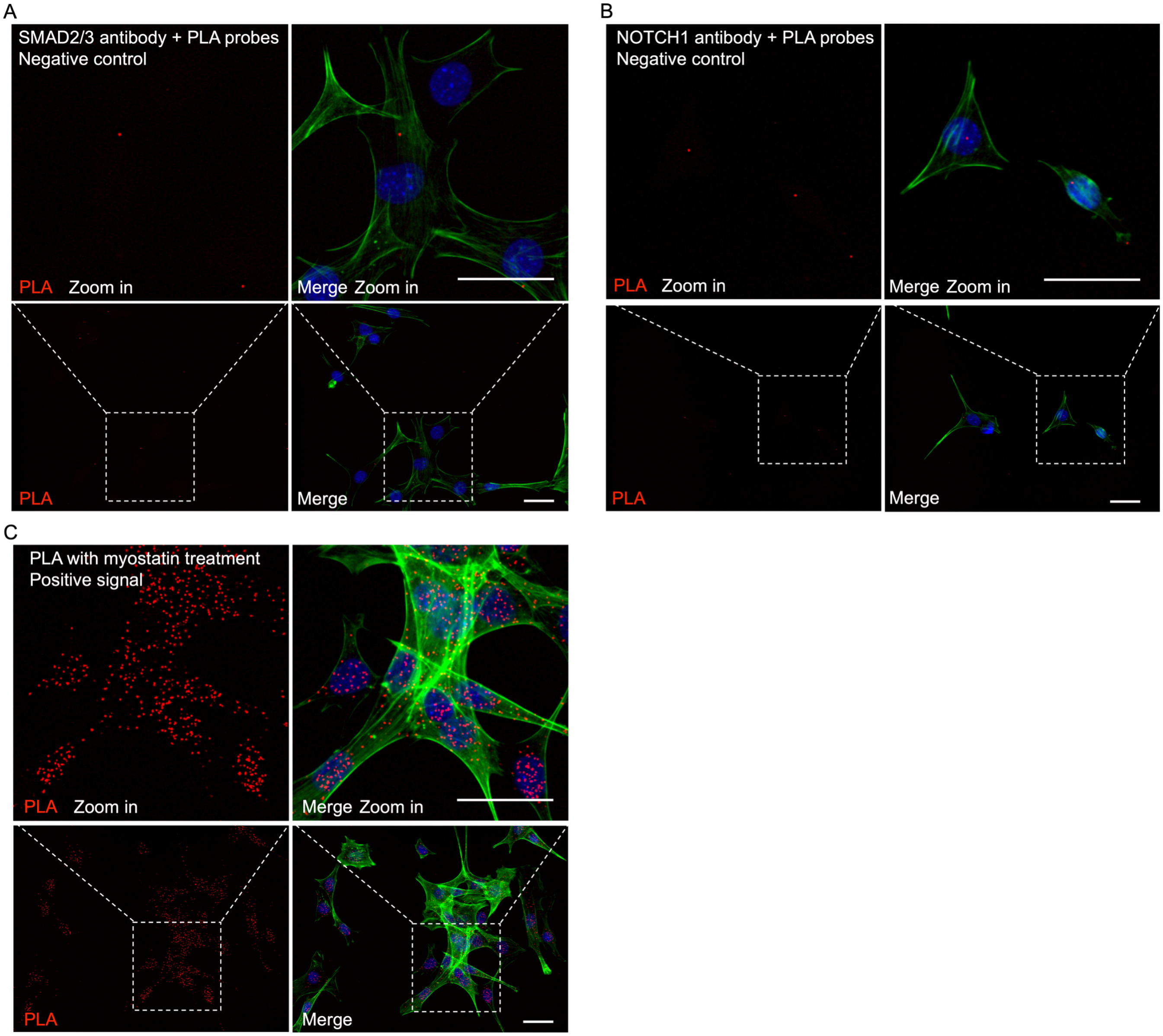

Supplementary Fig. 8. Interactions between Notch intracellular domain and SMAD2/3. A Negative control for in situ PLA using one primary antibody (SMAD2/3) and in **B** (NOTCH1) with two PLA probes. PLA signals (red), actin cytoskeleton (green) and nuclei counterstained with Hoechst 33342 (blue). Left panel, PLA signals alone. Right panel, PLA, actin cytoskeleton and nuclei merged. Scale, 50 µm. **C** Positive PLA interaction signal shown as comparison, as detected between SMAD and NOTCH1 after a 2h myostatin treatment at 4 µg/ml. Left panel, PLA signal alone. Right panel, PLA, actin cytoskeleton and nuclei merged. Scale bar, 50 µm. The images are direct control images for the experiment in Fig. 6B-C. The experiment was independently repeated three times.

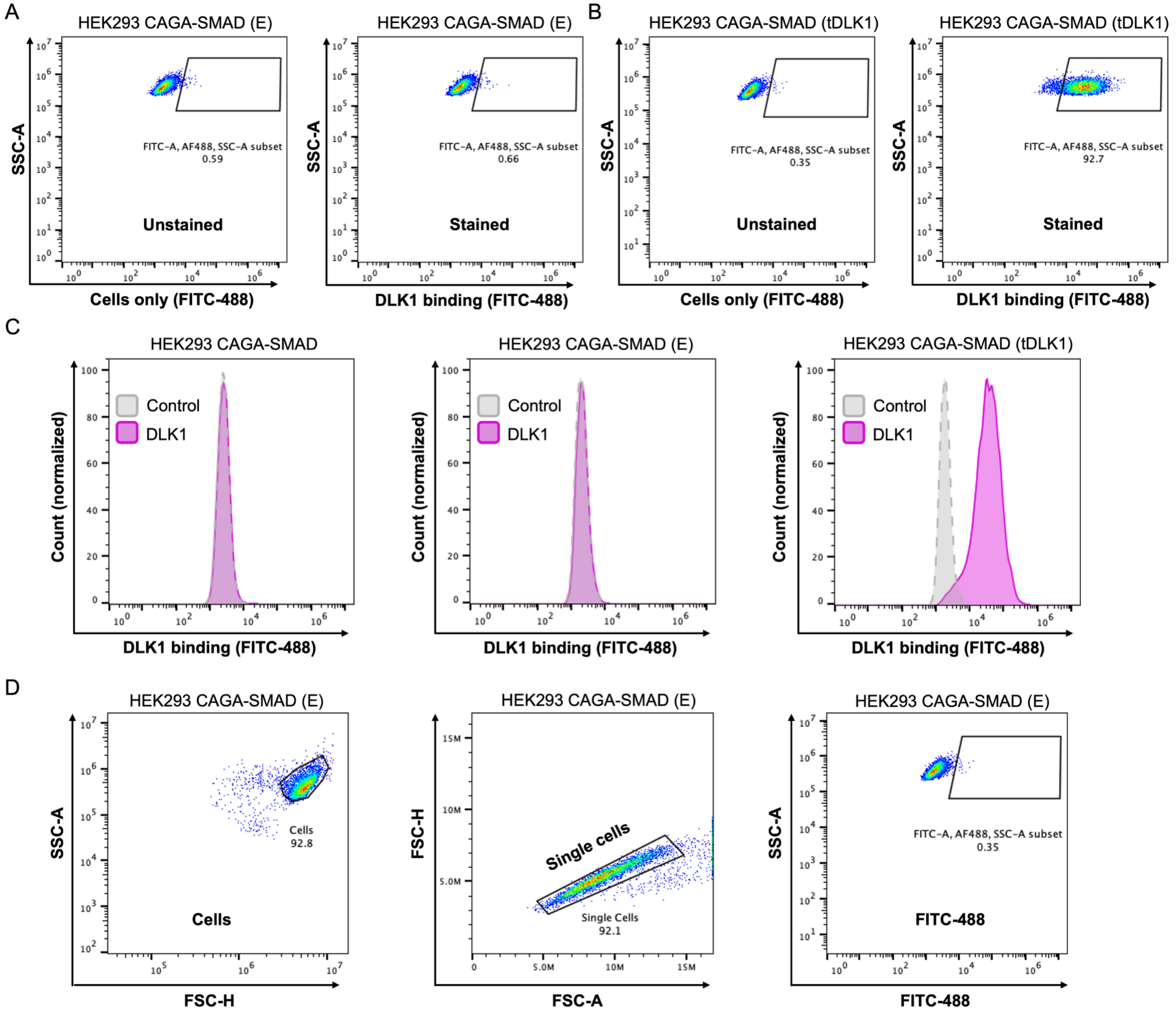

Supplementary Fig. 9. DLK1 surface expression and gating strategy. DLK1 is expressed on the surface of HEK293-$(CAGA)_{12}$ reporter cells after transfection**.** **A** Dot plot representations of flow cytometry using HEK293-$(CAGA)_{12}$ cells transfected with empty vector and stained by an anti-DLK1-488 antibody. The dot plots correspond to Supplementary Fig. 5D. **B** Dot plot representations of flow cytometry using HEK293-$(CAGA)_{12}$ cells transfected with DLK1 and stained by an anti-DLK1-488 antibody. The dot plots correspond to Fig. 5C and Supplementary Fig. 5D. **C** Histogram representations of HEK293-(CAGA)12 with or without empty vector or DLK1 and stained by an anti-DLK1-488 antibody. **D** Gating strategy for flow cytometry of DLK1 surface staining in HEK293 cells. The gating strategy corresponds to Supplementary Fig. 5D and Supplementary Fig. 9A-C.

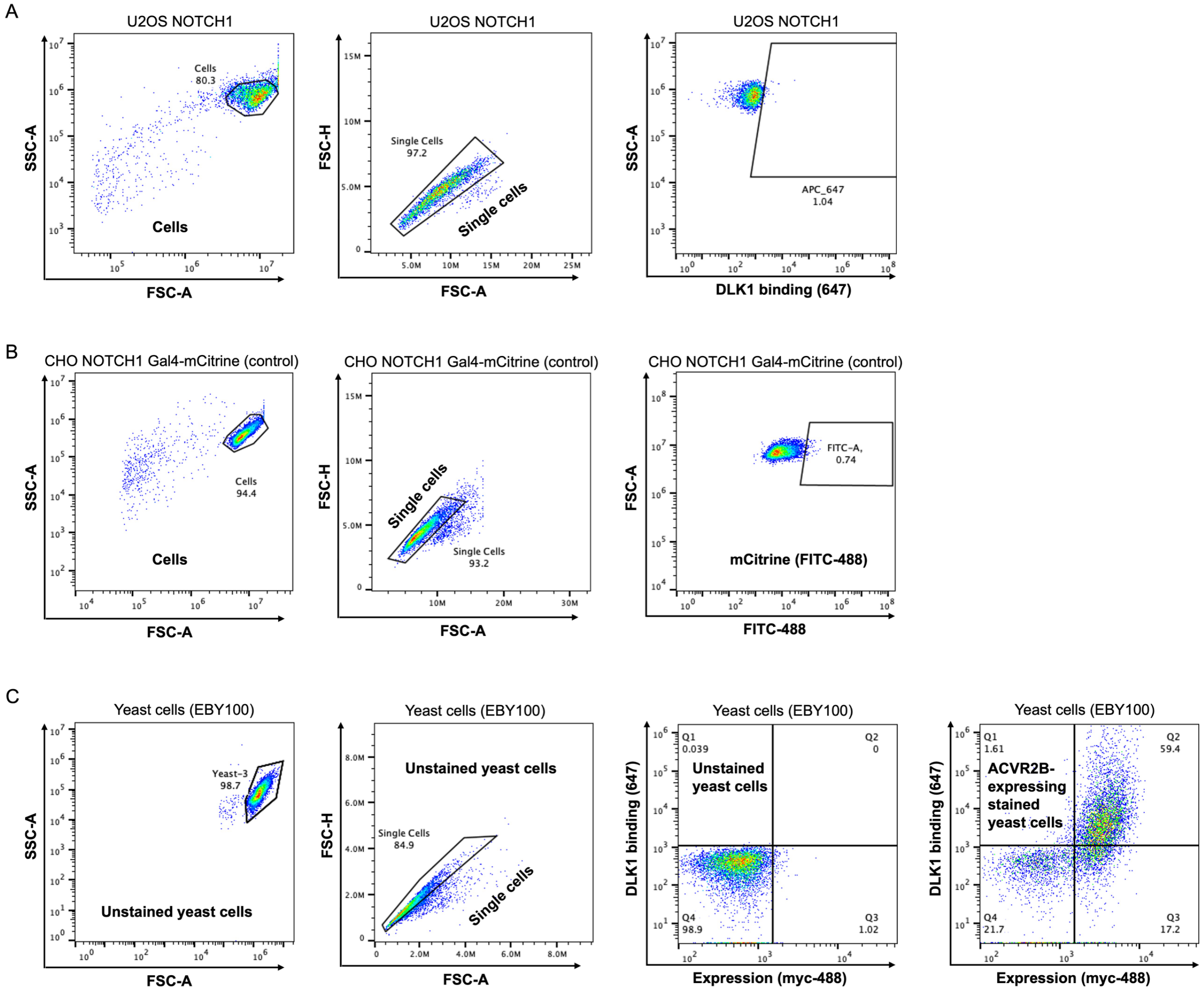

Supplementary Fig. 10. Gating strategies for U2OS, CHO and EBY100 yeast cells. A Gating strategy for flow cytometry of DLL4 and DLK1 surface staining in U2OS NOTCH1 cells. The gating strategy corresponds to Fig. 1C and Supplementary Fig. 1D. The sequential gating strategy is representative of all U2OS surface staining experiments. **B** Gating strategy for CHO NOTCH1 Gal4-mCitrine (FITC-488) reporter cells used for DLL4 and DLK1 activation and inhibition experiments. The gating strategy corresponds to Fig. 1D. **C** Gating strategy for EBY100 yeast cells expressing different TGF-β receptors. The gating strategy corresponds to Fig. 2E and Supplementary Fig. 2F.

Supplementary Table 1

Data collection	
Resolution (Å)[a]	35.89 - 2.67 (2.77 - 2.67)
Space group	C 1 2 1
Cell dimensions (Å; a, b, c);	120.98, 52.97, 97.99
Angle (°; α, β, γ)	90.00, 118.99, 90.00
Total reflections	52875<br>(5504)
Unique reflections	15578 (1550)
Completeness (%)	98.99 (96.33)
R_{merge}	0.04953 (0.6523)
R_{pim}	0.03153 (0.4046)
I/σ(I)	14.17 (1.71)
$CC_{1/2}$	0.999 (0.86)
Multiplicity	3.4 (3.6)
Wilson B-factor	74.31
**Refinement**	
Reflections used in refinement	15473 (1496)
R_{work}	0.2227 (0.4041)
R_{free}	0 0.2473 (0.4284)
Rmsd in bond lengths (Å)	0.006
Rmsd in bond angles (°)	1.13
Ramachandran favored (%)	92.90
Ramachandran outliers (%)	0
Rotamer outliers (%)	4.53
B- factor statistics (Å^2)	
Average B-factor	96.72
macromolecules	96.53
ligands	101.82
solvent	78.98

Supplementary Table 2

Protein-protein complex	Buried surface area (Å^2)	PDB ID
DLK1-ACVR2B	783.5	9D20
BMP2-ACVR2B	649.6	2H64
ACTIVIN A-ACVR2B	775.2	1S4Y
GDF11-ACVR2B	716.3	6MAC
