# Supplementary material for: Molecular mechanism of Activin receptor inhibition by DLK1
Source: Nat Commun. 2025 Jul 1;16:5976. doi: 10.1038/s41467-025-60634-3 (PMC12216052; doi:10.1038/s41467-025-60634-3)
Supplement: Supplementary file 2 — Description of Additional Supplementary Files [file 41467_2025_60634_MOESM2_ESM.pdf]

Description of Additional Supplementary Files

File name: Supplementary Data 1
Description: Gene fragments used for yeast display and primers used for cloning.
