# Supplementary material for: Molecular mechanism of Activin receptor inhibition by DLK1
Source: Nat Commun. 2025 Jul 1;16:5976. doi: 10.1038/s41467-025-60634-3 (PMC12216052; doi:10.1038/s41467-025-60634-3)
Supplement: Supplementary file 4 — Reporting Summary [file 41467_2025_60634_MOESM4_ESM.pdf]

nature portfolio

Corresponding author(s): Vincent C. Luca

Last updated by author(s): May 21, 2025

Reporting Summary

Nature Portfolio wishes to improve the reproducibility of the work that we publish. This form provides structure for consistency and transparency in reporting. For further information on Nature Portfolio policies, see our Editorial Policies and the Editorial Policy Checklist.

Statistics

For all statistical analyses, confirm that the following items are present in the figure legend, table legend, main text, or Methods section.

n/a	Confirmed	
☐	☒	The exact sample size (*n*) for each experimental group/condition, given as a discrete number and unit of measurement
☐	☒	A statement on whether measurements were taken from distinct samples or whether the same sample was measured repeatedly
☐	☒	The statistical test(s) used AND whether they are one- or two-sided<br>*Only common tests should be described solely by name; describe more complex techniques in the Methods section.*
☒	☐	A description of all covariates tested
☒	☐	A description of any assumptions or corrections, such as tests of normality and adjustment for multiple comparisons
☐	☒	A full description of the statistical parameters including central tendency (e.g. means) or other basic estimates (e.g. regression coefficient) AND variation (e.g. standard deviation) or associated estimates of uncertainty (e.g. confidence intervals)
☐	☒	For null hypothesis testing, the test statistic (e.g. *F*, *t*, *r*) with confidence intervals, effect sizes, degrees of freedom and *P* value noted<br>*Give P values as exact values whenever suitable.*
☒	☐	For Bayesian analysis, information on the choice of priors and Markov chain Monte Carlo settings
☒	☐	For hierarchical and complex designs, identification of the appropriate level for tests and full reporting of outcomes
☒	☐	Estimates of effect sizes (e.g. Cohen's *d*, Pearson's *r*), indicating how they were calculated

Our web collection on statistics for biologists contains articles on many of the points above.

Software and code

Policy information about availability of computer code

Data collection	X-ray data was collected remotely from Beamline 22-ID SERCAT at APS jusing NOMACHINE. Chromatography: Unicorn 7.1 (GE Healthcare). Biacore T200 software v3.0.(SRP). StepOnePlus Real-Time PCR system (Applied biosystems). BD Accuri C6 Plus Software (BD Biosciences). Phase contrast immunofluorescence images were acquired with a Keyence BZ-X710 microscope using a Nikon Plan Apo 10x objective and a Nikon S Plan Fluor 40x objective. Confocal images were imaged with a Leica SP8 laser scanning confocal microscope (Leica Microsystems GmbH, Germany). Luciferase data was recorded using GloMax Discover Software v3.2.3.
Data analysis	Data processing and structure refinement: XDS (Version March 15, 2019 BUILT=20190315), XDS (Version January 31, 2020, BUILT=20200131). Coot v0.8.9.1 EL, CCP4 v7.0.058, PyIMOL v.2.5.4, HKL-2000 v719.2, Prism 9 (GraphPad) V9.5.1, phenix v1.13-2998-000. Biacore T200 evaluation software v3.0. Excel (Microsoft). FlowJo 10.6.0. (BD). Phase contrast images were analyzed using BZ-X710LE analyzer software. Confocal images were analyzed with LAS X software version 3.7.4 (Leica Microsystems GmbH, Germany). Graphad Prism 10 Version 10.3.1 (464).

For manuscripts utilizing custom algorithms or software that are central to the research but not yet described in published literature, software must be made available to editors and reviewers. We strongly encourage code deposition in a community repository (e.g. GitHub). See the Nature Portfolio guidelines for submitting code & software for further information.

Data

Policy information about availability of data

All manuscripts must include a data availability statement. This statement should provide the following information, where applicable:

- Accession codes, unique identifiers, or web links for publicly available datasets
- A description of any restrictions on data availability
- For clinical datasets or third party data, please ensure that the statement adheres to our policy

X-ray crystallography data generated for the structure of DLK1 in complex with ACVR2B has been deposited in the Protein Data Bank under the accession code 9D20. In addition, the following crystallographic data were used in this study: PDB ID: 5UK5 (NOTCH1-JAG1), PDB ID: 4XBM (DLL1), PDB ID: 6MAC (ACVR2B-GDF11-ALK5), PDB ID: 5NH3 (ACVR2A), PDB ID: 1S4Y (Activin-ACVR2B), PDB ID: 2H64 (BMP2-ACVR2B), PDB ID: 5JI1 (GDF8), and PDB ID: 7MRZ (GDF11-ACVR2B). All data are included in the Supplementary Information or available from the authors. Unique reagents used in this study are available from the corresponding author on request. The raw numbers for all charts and graphs are available in a Source Data file. Source data are provided with this paper.

Research involving human participants, their data, or biological material

Policy information about studies with human participants or human data. See also policy information about sex, gender (identity/presentation), and sexual orientation and race, ethnicity and racism.

Reporting on sex and gender	This study does not involve human participants.
Reporting on race, ethnicity, or other socially relevant groupings	This study does not involve human participants.
Population characteristics	This study does not involve human participants.
Recruitment	This study does not involve human participants.
Ethics oversight	This study does not involve human participants.

Note that full information on the approval of the study protocol must also be provided in the manuscript.

Field-specific reporting

Please select the one below that is the best fit for your research. If you are not sure, read the appropriate sections before making your selection.

[x] Life sciences   [ ] Behavioural & social sciences   [ ] Ecological, evolutionary & environmental sciences

For a reference copy of the document with all sections, see nature.com/documents/nr-reporting-summary-flat.pdf

Life sciences study design

All studies must disclose on these points even when the disclosure is negative.

Sample size	No statistical method was used to determine sample size as it is not necessary for studies present in the manuscript. Samples used were sufficient for all parameters determined in this study. Cell staining experiments were performed with three replicates with at least two independent experiments. Crystallography data were collected for multiple crystals and the crystal with the best diffraction was used for structure determination.
Data exclusions	No data was excluded.
Replication	Experiments were repeated at least two times as described in the manuscript. Biochemical and biophysical measurements were performed using protein samples obtained from different protein purification preps. The replication attempts were successful.
Randomization	In cellular stainings and functional assays, there was not a specific order of measurement that would introduce a bias.
Blinding	Blinding is not relevant to the study as the output parameters are not subjective and therefore not subject to this form of bias.

Reporting for specific materials, systems and methods

We require information from authors about some types of materials, experimental systems and methods used in many studies. Here, indicate whether each material, system or method listed is relevant to your study. If you are not sure if a list item applies to your research, read the appropriate section before selecting a response.

Materials & experimental systems

n/a	Involved in the study	
	x	Antibodies
	x	Eukaryotic cell lines
x		Palaeontology and archaeology
x		Animals and other organisms
x		Clinical data
x		Dual use research of concern
x		Plants

Methods

n/a	Involved in the study	
x		ChIP-seq
	x	Flow cytometry
x		MRI-based neuroimaging

Antibodies

Antibodies used

anti-hPref1 Alexa Fluor 488 Conjugated antibody, Mouse monoclonal, Clone # 211309, R&D Systems Cat# FAB1144G, RRID:AB_3645938
anti-Myc-Tag (9B11) Mouse mAb Alexa Fluor® 488 Conjugated Cell Signaling Technology Cat# 2279, RRID:AB_2151849
anti Myosin Heavy Chain (MF20) mouse R&D Systems Cat# MAB4470, RRID:AB_1293549
anti-Notch1 Antibody (A-8) mouse monoclonal Santa Cruz Biotechnology Cat# sc-376403, RRID:AB_11149738
anti-Smad2/3 (D7G7) XP Rabbit mAb Cell Signaling Technology Cat# 8685, RRID:AB_10889933
Goat Anti-Human IgG Fc-AF647, Southern Biotech, Cat# 2048-31, RRID:AB_2795692
Goat anti-Mouse IgG (H+L) Highly Cross-Adsorbed Secondary Antibody, Alexa Fluor Plus 488, Thermo Fisher Scientific, Cat# A32723, RRID:AB_2633275
Rat anti-human IgG Fc (clone M1310G05) Alexa Fluor(R) 488, BioLegend, Cat# 410705, RRID:AB_2565783

Validation

anti-hPref1 Alexa Fluor 488 Conjugated antibody is validated by vendor and same clone as hPref1-647. Used in PMID: 32434947. In this study there was no staining of wild type cells that did not overexpress DLK1/Pref1.
- anti-Myc-Tag (9B11) Mouse mAb Alexa Fluor® 488 is validated for IF-IC, e.g. PMID:35316656, PMID:33606986, PMID:34825147
- anti Myosin Heavy Chain (MF20) mouse is recommended for Immunocytochemistry, Immunohistochemistry and WB by vendors. Used in multiple publications, e.g. PMID:38487591,PMID:37097817, PMID:36822206
- anti-Notch1 Antibody (A-8) mouse is validated for WB, IP, IF, ELISA. PMID: 38721633, PMID: 38923278, PMID: 36706917
- anti-Smad2/3 (D7G7) XP Rabbit mAb, PMID: 39138242 (WB), PMID: 31511510 (IF) , PMID: 28152139 (IHC-IF, WB).
- Goat Anti-Human IgG Fc-AF647 PMID: 20856794 (ELISA), PMID: 19825853 (WB), PMID: 20546303 (WB), PMID: 19188093 (SPR).
- Goat anti-Mouse IgG (H+L) Highly Cross-Adsorbed Secondary Antibody, PMID: 35680907(ICC/IF), PMID: 35379860 (ICC/IF), PMID: 35310916 (WB).
- Rat anti-human IgG Fc (clone M1310G05) Alexa Fluor(R) 488, the clone M1310G05 has been used in multiple publications e.g. PMID: 38172512(FC), PMID: 37011485 (FC).
We have tested all secondary antibodies alone without a primary antibody to confirm lack of non-specific binding.

Eukaryotic cell lines

Policy information about cell lines and Sex and Gender in Research

Cell line source(s)

C2C12 cells were from American Type Culture Collection (ATCC; Manassas, VA) (CRL-1772). HEK293T cells were a gift from Dr. Eric Lau (Moffitt Cancer Center, FL original commercial source ATCC, Cat# CRL-3216). Notch reporter cell lines CHO-K1 N1-Gal4 were a gift from Dr. M. Elowitz (California Institute of Technology, original commercial source Invitrogen, Cat# R71807). U2OS cells were a gift from Dr. Stephen Blacklow (original commercial source ATCC, Cat# HTB-96). HEK293-CAGA cells previously generated and published by Dr. Thomas Thompson (PMID: 22052913). Tni insect cells, Expression Systems, Cat# 94-002F.

Authentication

All cell lines were confirmed to have surface expression of the corresponding receptor using antibodies targeting each receptor and quantified using flow cytometry.

Mycoplasma contamination

All cells lines were tested for Mycoplasma prior to use, using the established kit by ATCC.

Commonly misidentified lines
(See ICLAC register)

No commonly misidentified cell lines were used in this study.

Plants

Seed stocks	not applicable
Novel plant genotypes	not applicable
Authentication	not applicable

Flow Cytometry

Plots

Confirm that:

- [x] The axis labels state the marker and fluorochrome used (e.g. CD4-FITC).
- [x] The axis scales are clearly visible. Include numbers along axes only for bottom left plot of group (a 'group' is an analysis of identical markers).
- [x] All plots are contour plots with outliers or pseudocolor plots.
- [x] A numerical value for number of cells or percentage (with statistics) is provided.

Methodology

Sample preparation	Mammalian cells were washed with DPBS and detached with trypsin-EDTA followed by centrifugation to remove residual trypsin-EDTA and staining with the corresponding antibody or protein was done in either 1% BSA in PBS or DMEM supplemented with 10% FBS for 1 h at 4 degrees celsius. Fc-tagged proteins were pre-mixed with anti-Fc IgG secondary antibodies before staining cells. For sorting DLK1, ACVR2B_GFP or GFP only expressing cells, a Sony SH800 sorter was used after a 1h antibody incubation with DLK1-PE-conjugated antibody or direct measurement of GFP fluorescence. Notch activation assays were performed by detaching CHO cells with trypsin-EDTA and resuspended in alpha-MEM supplemented with 10% FBS with 25 000 cells per well plated in 96-well plates on top of immobilized protein.
Instrument	BD Accuri C6 plus for all stainings and measurements. Sony SH800 for sorting mammalian cells.
Software	Data collection was performed using BD CSampler Plus Software v1.0.23.1 and analysis was done using FlowJo 10.8.1. and Sony SH800: cell sorter software v2.1.5.
Cell population abundance	Post sorting measurements of DLK1 over-expressing cells were confirmed to be over 90% positive for DLK1 staining compared to wild type cells. ACVR2B_GFP and GFP_only cells were sorted compared to non-transfected cells for positive GFP signal and post sort samples confirmed over 90% positive for GFP after sorting. After recovery, the cells were over 95% positive as determined by GFP signal.
Gating strategy	Cells were gated using the (log-log) FSC-A vs. SSC-A or FSC-H vs. SSC-A, followed by doublet discrimination using (linear-linear) FSC-A vs FSC-H, and the diagonal line for a ratio of 1:1 was gated for singlets. For CHO reporter cells, the gated single cells were then plotted using (log-log) FITC-A. vs. SSC-A. In the case of cell staining for receptor expression, the singlet cells were plotted using (log-log) FITC-A, PE-A, or APC-A vs. SSC-A depending on the fluorophore used. The ligand-binding population was defined as all cells within the gate that had a higher fluorescent signal compared to cells stained by secondary antibody alone. The gating strategies for all flow cytometry data can be found in Supplementary Fig. 9A-D and Supplementary Fig. 10A-C in the manuscript.

- [x] Tick this box to confirm that a figure exemplifying the gating strategy is provided in the Supplementary Information.
